# Testing the feasibility of eliciting preferences for health states from adolescents using direct methods

**DOI:** 10.1186/s12887-018-1179-7

**Published:** 2018-06-22

**Authors:** R. Trafford Crump, Ryan Lau, Elizabeth Cox, Gillian Currie, Julie Panepinto

**Affiliations:** 1 Department of Surgery, University of Calgary, 6601 7007 14 St SW, Calgary, AB T2V 1P9 Canada; 2 Department of Pediatrics, Medical College of Wisconsin, Milwaukee, WI USA; 3 Department of Pediatrics, University of Wisconsin, Madison, WI USA; 4 Departments of Paediatrics and Community Health Sciences, University of Calgary, Calgary, AB Canada

**Keywords:** Adolescents, Survey, Health states, Preferences, Psychometrics

## Abstract

**Background:**

Measuring adolescents’ preferences for health states can play an important role in evaluating the delivery of pediatric healthcare. However, formal evaluation of the common direct preference elicitation methods for health states has not been done with adolescents. Therefore, the purpose of this study is to test how these methods perform in terms of their feasibility, reliability, and validity for measuring health state preferences in adolescents.

**Methods:**

This study used a web-based survey of adolescents, 18 years of age or younger, living in the United States. The survey included four health states, each comprised of six attributes. Preferences for these health states were elicited using the visual analogue scale, time trade-off, and standard gamble. The feasibility, test-retest reliability, and construct validity of each of these preference elicitation methods were tested and compared.

**Results:**

A total of 144 participants were included in this study. Using a web-based survey format to elicit preferences for health states from adolescents was feasible. A majority of participants completed all three elicitation methods, ranked those methods as being easy, with very few requiring assistance from someone else. However, all three elicitation methods demonstrated weak test-retest reliability, with Kendall’s tau-a values ranging from 0.204 to 0.402. Similarly, all three methods demonstrated poor construct validity, with 9–50% of all rankings aligning with our expectations. There were no significant differences across age groups.

**Conclusions:**

Using a web-based survey format to elicit preferences for health states from adolescents is feasible. However, the reliability and construct validity of the methods used to elicit these preferences when using this survey format are poor. Further research into the effects of a web-based survey approach to eliciting preferences for health states from adolescents is needed before health services researchers or pediatric clinicians widely employ these methods.

**Electronic supplementary material:**

The online version of this article (10.1186/s12887-018-1179-7) contains supplementary material, which is available to authorized users.

## Background

Measuring adolescents’ preferences for health states can play an important role in evaluating the delivery of pediatric healthcare. Health states describe a scenario that an individual may experience at a particular point in time [1]. The scenario is comprised of attributes that define physical and mental functional abilities, and the severity of symptoms. These attributes may be real – that is, those currently being experienced by the individual. Alternatively, these attributes may be hypothetical, where the individual is asked to imagine what it would be like to experience the scenario. The choice between using real or hypothetical attributes depends on the research objectives [1]. For example, when screening or monitoring an individual patient’s health, real attributes are used and assessed. When developing a population health status index, hypothetical attributes may be used. Each attribute used in a health state is described using levels that lie on a continuum between perfect health and death (e.g., “no pain”, “moderate pain”, “severe pain”). One level per attribute is used to describe a health state [2].

By systematically altering the levels used to describe the attributes, different health states can be formed. The extent to which an individual desires one health state over another – referred to as a preference in this study – can then be measured. It is common practice to measure preferences for health states in adults [3]. A systematic review identified 344 studies eliciting health and health care preferences from adults using direct methods [4]. The visual analogue scale, time trade-off and standard gamble are all common direct methods used to measure preferences [5]. Discrete choice experiments are also commonly used to elicit preferences for health and health states [6].

Despite its prevalence in adults, measuring preferences for health states from adolescents is far less common. In part, this rarity is due to two challenges. The first challenge is logistical. Previous studies have observed difficulty in identifying, recruiting, and retaining adolescents in clinical studies [7, 8]. These studies have reported several reasons for this: 1) receiving approval from institutional review boards, 2) protective parents not wanting to burden their adolescents, and 3) keeping adolescents sufficiently engaged to maintain their motivation in study participation [7, 9]. Consequently, the cost of including adolescents participants can be prohibitive. Some researchers have reported success by using the internet (e.g., world wide web, email, social networks) to overcome these logistical challenges [7, 8, 10].

The second challenge to measuring preference for health states from adolescents is conceptual [11, 12]. It can be difficult to develop health states that reflect the changing physical, social, and psychological factors that adolescents experience as they mature [11]. Adolescents also have limited frames of reference – that is, experiences living in different states of health – which can threaten the validity and reliability of eliciting their preferences [13]. When asked about their preferences for health states that they have not experienced, such as hypothetical or future health states, adolescents of all ages have demonstrated seemingly illogical risk taking [12].

As a result of the logistical and conceptual challenges identified above, adolescents’ preferences for health states are often elicited from adults or proxies, such as parents or care givers [13, 14]. Using proxy preferences, however, is not without its own shortcomings. Some argue that proxies fail to accurately assess the importance of certain unobservable health domains for adolescents, such as social or emotional impairments [13, 15]. For example, health problems that impact body image or ability to socialize with peers can be of far greater importance to adolescents than adults [11]. As a result, significant differences in preferences for health states elicited from adolescents and their parents have been observed [16, 17].

Given the challenges associated with proxies, measuring preferences directly from adolescents is of critical importance, particularly in defining the value of specific interventions in pediatric healthcare [18]. Before doing so, however, we need to establish the feasibility of recruiting adolescent participants and the appropriate elicitation methods for measuring health state preferences from this population. A recent systematic review of studies that directly measured preferences in adolescents observed that only 26 out of 74 studies in the past 25 years have reported some form of feasibility, reliability, or validity for commonly used elicitation methods [19]. To move the field forward, there is a need to better understand how these elicitation methods work with adolescents.

Therefore, the primary aim of this study was to test the feasibility of eliciting adolescents’ preferences for health states using a web-based survey approach. If this was feasible, it’s secondary aims were to 1) test the reliability and validity of different commonly used direct elicitation methods, and 2) assess whether there were differences across age groups in the reliability and validity of using these methods. This was an exploratory study, with no hypotheses a priori.

## Methods

### Developing the web-based survey

The research team first conceptualized and designed the survey on paper, which went through several reviews and iterations (Additional file 1). Based on this initial design, the survey was converted to a web application using Qualtrics software (Qualtrics LLC. Provo, UT) by the University of Wisconsin Survey Center (Madison, WI, USA). The Qualtrics software enabled the survey to be compatible on any web-enabled device (e.g., computer, tablet, mobile phone).

### Developing the health states

The health states constructed for the survey used in this study were based on the Patient-Reported Outcomes Measurement Information System’s (PROMIS) Pediatric Profile [20]. The PROMIS Pediatric Profile measures seven domains of child health: anxiety, depression, fatigue, pain intensity, pain interference, physical function, and peer relationships. For each domain, the respondent is asked to rank how commonly a symptom or functional limitation impacted their life over the last seven days. These are ranked using a five-point Likert scale, ranging from “Never” to “Almost Always”.

Several modifications were made to the PROMIS Pediatric Profile in order to reduce the number of health states used in this study. First, only one item per domain was used to describe the health state. Second, the middle ranking, “Sometimes”, was not used to describe any items. Third, the pain intensity domain was dropped because of its descriptive overlap with the pain interference domain.

**Fig. 1:**
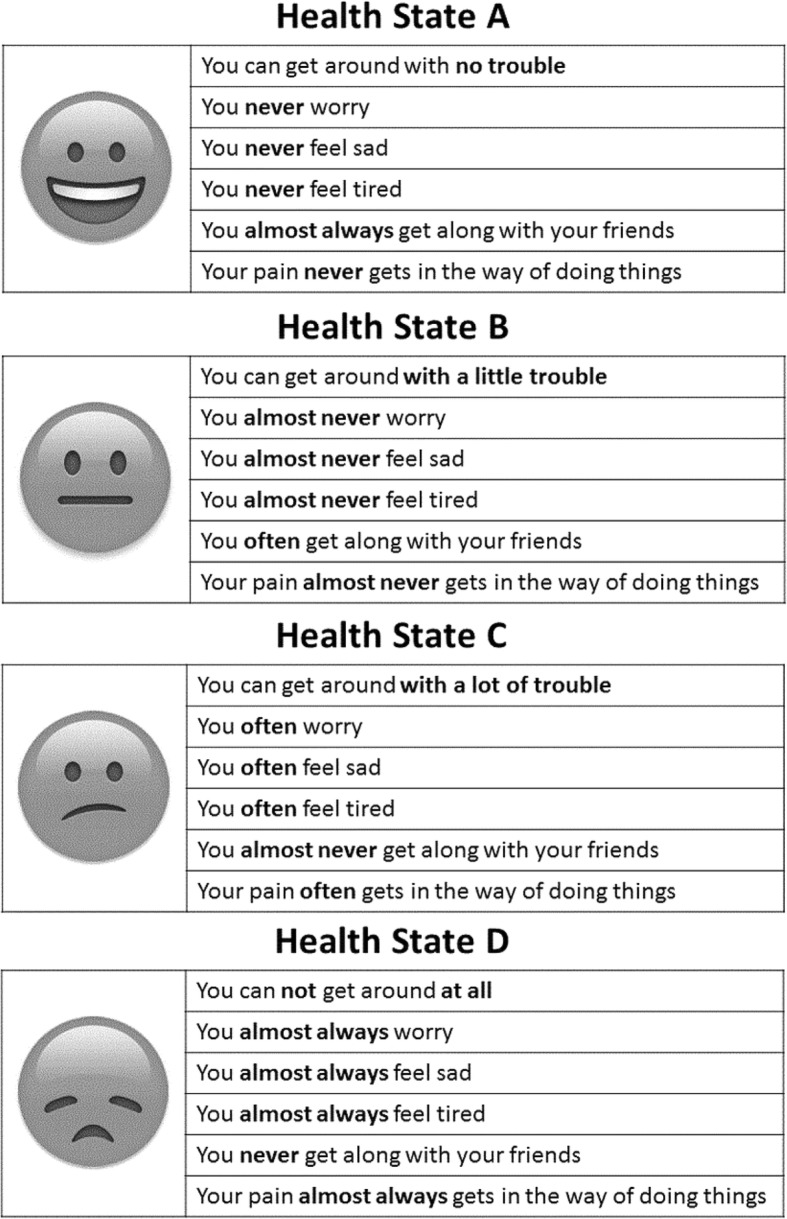
The four health states used in the survey

After these modifications, four hypothetical health states were established, each comprised of six attributes (Fig. 1). The health states were generically labeled A through D. Two of the health states represented the most extreme descriptions: Health State A described “perfect health”, and Health State D described “worst health”. The other two, Health States B and C, described relatively less extreme states. This provided a rational order, which we could later exploit to test validity. A graphic icon was used to help respondents more easily identify the health states in the web-based survey, similar to what has been done previously [21].

### Measuring preferences for the health states

The survey was divided into three main sections, each related to one of the three direct elicitation methods under study: 1) visual analogue scale, 2) time trade-off, and 3) standard gamble. The selection of these direct elicitation methods was based on a review of those that have demonstrated feasibility, reliability, and validity with adolescents [19]. An example of how each method was presented to the respondent is provided as a supplemental appendix. After each of these sections, respondents were asked to rate the difficulty of the method they had just completed using a four-point Likert scale (ranging from 1 = “no difficulty completing” to 4 = “completed with a lot of trouble”) and whether they required assistance to complete the method.

Section one involved the visual analogue scale. Participants were presented with the four health states (i.e., Health States A-D) in random order and asked to place them along a vertical scale ranked from “best”) to “worst”. Participants were first instructed to place the health state they favored the most at the top of a scale, and the one they favored the least at the bottom of the scale. They were then instructed to place the remaining health states in-between the best and worst health states, based on how much they favored each relative to the best/worst anchors.

Section two involved a modified version of the time trade-off. Participants were presented with two health states. The first health state was always perfect health (i.e., Health State A). The second was one of the relatively less healthy states (i.e., Health State B, C, or D). Participants were told that they could live for 60 years in the relatively less healthy state, or live in Health State A for fewer than 60 years. Participants were presented with a sliding scale ranging between 0 and 59 and instructed to slide the scale to indicate the fewest number of years they would be willing to live in Health State A. They could slide the scale around and see the number of years change, as though it were an iterative process. This task was completed for all three of the relatively less healthy states. This modified version of the time trade-off was based on similar work used with adolescents by Moodie et al. [21] and was necessary for ease of use in a web application.

Section three involved a modified version of the standard gamble. Participants were presented with three health states. The first was always perfect health (i.e., Health State A) and the second was always worst health (i.e., Health State D). The third health state was either one of the relatively moderate health states (i.e., Health State C or D). Participants were first instructed that they had the choice between:

1. a 100% chance of living in Health State A and a 0% chance of living in Health State D, or
2. a 100% chance of living in one of the relatively moderate health states.

This choice was intentionally set-up with a dominant decision (i.e., option #1). Provided the participants chose this option, the chance of living in Health State A was systematically reduced (and, by corollary, the chance of living in Health State D was systematically increased). This process continued until the participants switched their preference to option #2. This task was completed for both the relatively moderate health states. This modified version of the standard gamble was based on similar work used with adolescents by Juniper et al. [14] and was necessary for ease of use in a web application.

### Piloting the survey

This survey format was piloted with high school students from Dodgeville High School (Dodgeville, WI, USA). It took the students between 12 to 18 min to complete the survey, which was within an acceptable range. Based on the quantitative and qualitative feedback received from the pilot, the survey format and some of the instructions were revised for the development of the final version used in this study. For example, wording around the instructions for the time trade-off changed based on feedback from the students.

### Study sample

Adolescents from the general population in the United States were sampled by the University of Wisconsin Survey Center. The sample was derived from a standing panel of households across the United States that had previously self-identified as having adolescents and willing to participate in web surveys. A sample of 100 adolescents was desired to test the feasibility of conducting this kind of survey. A response rate of 30% was expected. Consequently, a convenience sample of 300 parents from the University of Wisconsin Survey Center’s panel of households were sent emails soliciting their adolescents’ interest in participating in this study.

To be eligible for this study, adolescents had to be under the age of 18, attending school, able to read and understand English, and have access to a web-enabled device. Adolescents were excluded if they did not meet the inclusion criteria. Participants were given an incentive of $1 to complete the initial survey and an additional $1 if they completed the repeat survey. As this was a feasibility study – primarily aimed at testing the viability of conducting a survey of this nature via the internet – no attempt was made to have this sample be representative of any particular population.

At the end of the survey (i.e., the initial survey), participants were asked if they would be willing to re-take the survey at a later time. Those that agreed were contacted a week later via email and asked to complete the identical survey (i.e., the repeat survey). The data were linked by the University of Wisconsin Survey Center in order for the research team to perform the comparative analysis between the two surveys.

The survey was anonymous and no data personally identifying the participants were provided to the investigators. This study was approved by the Institutional Review Board of the Adolescents’ Hospital of Wisconsin which waived the need for written consent.

### Data analysis

Descriptive statistics were used to characterize the sample for both the initial and repeat surveys. Differences between samples were tested using the student t-test or Pearson’s Chi-square, as appropriate. All tests were two-tailed, and the findings were considered statistically significant if the *p*-value was < 0.05.

In order to address this study’s primary aim, the feasibility of each elicitation method was conceptually defined as participants’ willingness and ability to complete it [22]. “Willingness” was operationalized by measuring the number of participants who skipped or did not complete the elicitation method. “Ability” was operationalized by eliciting participants’ feedback as to the difficulty of the method and their need for assistance. Methods with higher completion rates, with greater number of participants characterizing it as being “easy”, and requiring less help from others were considered relatively more feasible.

Two analytic approaches were taken to measure the reliability and validity of the elicitation methods – the first of this study’s secondary aims. Reliability was conceptually defined as the method’s ability to reproduce similarly ranked results over multiple points of administration [22]. For those participants who completed the initial and repeat surveys, a test-retest comparison was possible. Therefore, reliability was measured separately for each elicitation method using the Kendall tau-a correlation [23]. Values from the Kendall tau-a can be interpreted as: 1 = complete agreement, − 1 = complete disagreement, 0 = random. Methods with higher Kendall tau-a values were considered relatively more reliable.

The health states used in this survey were intentionally constructed to have an expected order: from most to least preferable (i.e., Health State A through D). Construct validity was conceptually defined by how frequently participants’ respective ordering matched with this expected order. This definition is similar to how it has been defined in the health state valuation literature [22]. Operationally, it was measured by the frequency with which the orders matched, for each elicitation method. Methods with higher rates of expected ordering were considered more valid.

Previous studies eliciting preference for health states from adolescents have observed differences in the feasibility and reliability of responses across age groups [14, 24]. Preference elicitation methods tend to have higher completion rates and more consistent responses in relatively older adolescents. Given these observations, then, we tested whether there were significant differences in terms of feasibility, reliability, and validity across age groups. To create relatively equal sub-groups, we categorized participants based on their age into one of six sub-groups: 12 years and under, 13, 14, 15, 16, and 17–18 years of age.

## Results

A total of 293 solicitation emails were sent out and delivered (7 emails were undeliverable). From those, 168 adolescents started and completed the initial survey (response rate = 57%). Of these participants, 23 were dropped because they were older than 18 and one was dropped because they had already graduated from high school. Thus, there were a total of 144 participants in this study (participation rate = 49%). Details regarding the non-participants were not available for comparison. As detailed in Table 1, the age of participants in the initial survey ranged from 10 to 17 years, with a mean of 14.5 years. The school grade of participants in the initial survey ranged from 4 to 12, with the mode being the 9th grade. A total of 103 (72%) participants repeated the survey one week after the initial survey. There were no statistically significant differences in terms of age or grade between those who completed the initial and repeat surveys.

**Table 1:** Sample characteristics for the initial and repeat surveys

	Initial survey n (%)	Repeat survey n (%)	Test for differences
Total sample	144	103	
Age			
10	3 (2)	2 (2)	*t* = 0.027 *p* = 0.978
11	2 (1)	0 (0)	
12	13 (9)	10 (10)	
13	26 (18)	20 (19)	
14	27 (19)	23 (22)	
15	28 (19)	18 (17)	
16	18 (12)	9 (9)	
17	27 (19)	19 (18)	
18	0 (0)	2 (2)	
School grade			
4	1 (1)	0 (0)	Chi^2^ = 2.886 *p* = 0.941
5	5 (3)	3 (3)	
6	5 (3)	6 (6)	
7	15 (10)	10 (10)	
8	23 (16)	13 (13)	
9	34 (24)	24 (23)	
10	19 (13)	18 (17)	
11	22 (15)	14 (14)	
12	20 (14)	15 (15)	

### Feasibility

We assessed the feasibility of the preference elicitation methods based on the number of elicitation methods that were completed. As detailed in Table 2, all participants (*n* = 144; 100%) completed the visual analogue scale, followed by the standard gamble (*n* = 139; 97%), and the time trade-off (*n* = 109; 76%). A similar pattern was observed for those methods characterized as being “very easy”: 82% (*n* = 118) for the visual analogue scale, 76% (*n* = 110) for the standard gamble, and 66% (*n* = 95) for the time trade-off. A similar number of participants, 16–17%, needed assistance with completing the methods. Of those that needed assistance, many sought help from a parent or friend.

**Table 2:** Feasibility assessment includes competition rankings, reported difficulty of each preference elicitation method

*n* = 144	Visual analogue scale n (%)	Time trade-off n (%)	Standard gamble n (%)
Participants who completed all rankings	144 (100)	109 (76)	139 (97)
Difficulty of method			
Very easy	118 (82)	95 (66)	110 (76)
Not too easy / Not too difficult	25 (17)	31 (22)	30 (21)
Very difficult	1 (1)	6 (4)	3 (2)
Missing	0 (0)	12 (8)	1 (1)
Needed help completing the method			
Yes	24 (17)	23 (16)	23 (16)
No	119 (83)	109 (76)	120 (83)
Missing	1 (1)	12 (8)	1 (1)
Who provided help			
Parent	13 (54)	9 (39)	7 (30)
Sibling	1 (4)	5 (22)	5 (22)
Friend	8 (33)	7 (30)	8 (35)
Other	1 (4)	2 (9)	3 (13)
Missing	1 (4)	0 (0)	0 (0)

For the visual analogue scale completion rates ranged from 74% (for the 17–18 age group) to 96% (for the 13-year-old age group), however the differences were not statistically significant (Chi^2^ = 6.15; *p* = 0.292). For the time trade-off, completion rates ranged from 67% (for both the 12-and-under age group and the 14-year-old age group) to 85% (for the 17–18 age group), but again these differences were not significant (Chi^2^ = 4.53; *p* = 0.476). Finally, for the standard gamble, completion rates ranged from 88% (for the 13-year-old age group) to 100% (for all other age groups, except the 14-year-old age group), and these differences were not significant (Chi^2^ = 9.57; *p* = 0.088).

### Reliability

The results of test-retest analysis are provided in Table 3. None of the methods demonstrated particularly strong or weak agreement between the initial and repeat surveys. Kendall’s tau-a values varied within each method, ranging from 0.204 to 0.402, indicating weak agreements between the initial and repeat survey.

**Table 3:** Test-retest reliability as assessed by Kendall’s tau-a scores

	Kendall’s tau-a
Visual analogue scale (*n* = 79)	
Health State A	0.20
Health State B	0.40
Health State C	0.29
Health State D	0.30
Time trade-off (*n* = 68)	
Using Health State B	0.38
Using Health State C	0.22
Using Health State D	0.22
Standard gamble (*n* = 83)	
Using Health State B	0.37
Using Health State C	0.25

The proportion of participants who repeated the survey ranged from 47% in the 16-year-old age category to 80% in the 12-and-under age category. Some differences were observed when comparing the Kendall’s tau-a across age categories, but in no significant or discernable pattern. For example, those in the 17–18 age category had higher Kendall’s tau-a values for the visual analogue scale (ranging from 0.242–0.528), but those in the 16-year-old age category had the lowest (ranging from − 0.133-0.267). For the time trade-off and the standard gamble, the 14-year-old age category had the highest Kendall’s tau-a values with the narrowest range (range from 0.319–0.727 for the time trade-off; 0.506–0.524 for the standard gamble).

### Construct validity

Construct validity was measured by the frequency with which health states were ranked in accordance with what was expected (i.e., A ranked higher than B, B ranked higher than C, etc.), and fully detailed in Table 4. For the visual analogue scale, 65% (*n* = 93) participants gave Health State A the highest ranking. However, only 45% (*n* = 65) gave Health State D the lowest ranking. A minority (38%) of participants ranked all the health states as expected.

**Table 4:** The frequency that health states were ranked as expected

	Frequency (%)
Visual analogue scale	*n* = 144
Health State A highest ranking	93 (65)
Health State B second highest ranking	57 (40)
Health State C second lowest ranking	60 (42)
Health State D lowest ranking	65 (45)
All ranked as expected	55 (38)
Time trade-off	*n* = 109
Health State B highest ranking	23 (21)
Health State C middle ranking	10 (9)
Health State D lowest ranking	24 (22)
All ranked as expected	10 (9)
Standard gamble	*n* = 139
Health State B highest ranking	105 (76)
Health State C lowest ranking	89 (64)
All ranked as expected	70 (50)

Because Health State A was used as the reference state in the time trade-off exercise, only the rankings for Health States B, C and D could be measured. Compared to the other methods, the time trade-off had the worst concordance with the expected ranking. No more than 22% of participants ranked any of the health states as expected, and only 9% ranked all of them as expected.

The standard gamble used Health States A and D as the reference states, thus only the rankings of B and C could be measured. The majority of participants ranked Health States B and C as expected. Half of the participants ranked the two health states as expected.

When comparing across age groups, there were no statistically significant differences in terms of the number of health states that were ranking according to expectations. The visual analogue scale (Chi^2^ = 4.03; *p* = 0.545), time trade-off (Chi^2^ = 9.03; *p* = 0.108), and standard gamble (Chi^2^ = 3.22; *p* = 0.666) all performed similarly across the age groups.

## Discussion

The primary purpose of this exploratory study was to assess whether it was feasible to elicit adolescents’ preferences for health states using a web-based survey approach. Of the 144 adolescents who initiated the survey, all or nearly all of them completed the visual analogue scale and standard gamble, three-quarters of them completed the time trade-off. A majority of these participants said that they found the elicitation methods easy, with approximately 16% seeking assistance from someone else. Based on these results, we believe that a web-based approach for preference elicitation is feasible. Participants were successfully recruited, engaged, and completed in the survey.

One of the secondary purposes of this study was to test the reliability and validity of the visual analogue scale, time trade-off, and standard gamble. In terms of reliability, none of the methods performed particularly well. The range of Kendall tau-a values all indicated weak agreement between the initial and repeat surveys. In terms of construct validity, the number of participants ranking health states per our expectations for all three elicitation methods was also poor. The performance of the methods (i.e., the standard gamble being the relatively-best performing method and the visual analogue scale the relatively-worst) was opposite of our assumptions. However, the standard gamble also had the fewest comparisons (two) and, thus, the fewest opportunities for rankings to be discordant from our expectations.

Results of the reliability and construct validity have us questioning whether the participants fully understood the methods and what was being asked of them. This is even more concerning when taken with the observation that so many participants ranked these methods as being very easy. This may be an issue with the preference elicitation methods. Previously studies employing these methods and publishing their psychometric properties have reported mixed results [19]. Or, it may have more to do with the web-based survey format. While web-based health studies have been previously used with adolescents samples [25, 26], we cannot find sufficient evidence as to the possible effect of this survey format on responses. The use of web-based surveys for adolescent health studies is an area that has gone largely un-studied and will require more research if this mode of administration is to become more prevalent with this demographic.

The other secondary purpose of this study was to test for differences across age groups in the reliability and construct validity of the elicitation methods. None of the methods demonstrated any significant differences across age groups in any of the measured outcomes, though this could be a bi-product of our small age sub-groups. These observations are supported by previous studies that have compared differences in preference elicitation exercises for health states across age groups. In a study by Juniper et al., participants ranging from 7 to 17 years old were asked to use a feeling thermometer (a form of the visual analogue scale) and the standard gamble, amongst other preference measurement methods. The authors observed that all but the very youngest participants could comprehend and use the feeling thermometer, and those with a sixth-grade reading level (i.e., approximately 11–12 years of age) and above could comprehend and use the standard gamble [14]. Similarly, in using the standard gamble with participants between 10 and 18 years of age, Brunner et al. reported that all participants had a good understanding of the methods, but cautioned in their conclusion that results from younger participants should be carefully examined [24].

This study has several shortcomings that may limit its generalizability. First, we cannot verify the age of the participant. The initial solicitation was sent to households that were known to have adolescents under the age of 18. However, there was no way to verify (other than the question regarding age and school grade) that it was, in fact, the child who completed the survey. This is a risk for all internet-based surveys, which are becoming increasingly more popular to cost-effectively collect data from large segments of the populations [27]. Second, we had technical limitations to construct fully interactive time trade-off and standard gamble methods. While our modified versions of these methods have been used in peer-reviewed studies previously, we cannot say definitively how those modification altered our results regarding the reliability and construct validity. Third, stratifying our sample into age sub-groups resulted in small sub-sample sizes (~ 25 participants per sub-group), which may influence the results. Hence why this sub-group analysis was left as secondary aim. Larger, more representative studies would be needed to test differences in the performance of these elicitation methods across age groups with more confidence.

Despite these limitations, the results from this feasibility study demonstrate that administering a survey eliciting preferences for health states from adolescents via the web is feasible. These results will be relevant to those health services researchers trying to develop preference weights for patient-reported outcome instruments so that they may elicit preferences using indirect methods [28]. They may also be relevant to clinicians wanting to incorporate their younger patients’ perspectives into their clinical decision making [15].

## Conclusion

To conclude, using a web-based survey format to elicit preferences for health states from adolescents is feasible. However, the reliability and construct validity of the methods used to elicit these preferences when using this survey format are poor. Further research into the effects of a web-based survey approach to eliciting preferences for health states from adolescents is needed before health services researchers or pediatric clinicians widely employ these methods.

## Additional file

Additional file 1: Survey instrument. Survey instrument used for this study. (DOCX 17 kb)

## Abbreviations

PROMIS: Patient-Reported Outcomes Measurement Information System
