# Supplementary material for: Testing the feasibility of eliciting preferences for health states from adolescents using direct methods
Source: BMC Pediatr. 2018 Jun 22;18:199. doi: 10.1186/s12887-018-1179-7 (PMC6015455; doi:10.1186/s12887-018-1179-7)
Supplement: Supplementary file 1 — Survey instrument. Survey instrument used for this study. (DOCX 17 kb) [file 12887_2018_1179_MOESM1_ESM.docx]

Q1 Welcome Thank you for agreeing to be part of this study. We want to know how best to ask youths their opinions on different health states. Measuring these opinions are important. With them, we can better know the kind of health care youths like, without having to ask adults.

Q2 This survey has 5 sections. You can see where you are by the bar at the top of the screen. Specific instructions are given before each section. Please read and follow them carefully. There are no right or wrong answers to these questions. Your own opinion is what counts. It's ok if you don't understand a question.  If you don’t understand a question, skip it and move on to the next.  You’ll be given a chance to explain why you didn’t understand the question at the end of every section.

Q3   Throughout this survey, you’ll be asked about 4 different health states. Health states describe your ability to work at school, play with friends, and sleep. Health states also describe how you feel about yourself and how you think others feel about you. These health states are imaginary, but think of yourself having to live your life as it describes. There&#39;s no need to memorize the health states, you&#39;ll see them again throughout this survey.

Q86 Health State A through Health State D

Q10 We’d like to know about how you feel about the health states. In this section, please: 1. Place the health state you think is the best at the top. 2. Place the health state you think is the worst at the bottom.  3. Slide the remaining two health states along the scale depending on how much you like them compared to the best and worst.

BEST

______ Image:Health state d s (2)

______ -- (18)

______ Image:Slide5s (4)

______ -- (19)

______ -- (20)

______ -- (21)

______ Image:Slide2s (1)

______ -- (22)

______ -- (23)

______ Image:Slide4s (3)

WORST

Q11a We'd like to know how difficult you found the last section. Overall, I found the last section:

- Very Easy (1)
- Not too easy but not too difficult (2)
- Very Difficult (3)

Q11b Did you need help with the last section?

- Yes (1)
- No (2)

Q11c Write any comments you may have about the last section here:

Q11d If you needed help with the last section, who helped you?

- Parent (1)
- Brother or Sister (2)
- Friend (3)
- Other (4)

Q13 InstructionsWe'd like to know how you feel about Health State A compared to other health states. In this section, please:1. Imagine that ... You could live in the described health state for 60 more years.  OR  You could live in Health State A, but for less than 60 years. 2. Slide the marker on the time-line to indicate the fewest number of years you'd be willing to live in Health State A instead of the one described.

Q14 Imagine that you are in Health State D and you have 60 years left to live. We are interested to know whether you would be prepared to live for less than 60 years if your health could be excellent as in Health State A.  Please slide the marker to the shortest time you would accept Health State A instead of 60 years in Health State D.  This is the amount you have chosen to live in excellent health. You are giving up the rest of the 60 years.

______   Number of Years (1)

Q15 Imagine that you are in Health State C and you have 60 years left to live. We are interested to know whether you would be prepared to live for less than 60 years if your health could be excellent as in Health State A.  Please slide the marker to the shortest time you would accept Health State A instead of 60 years in Health State C.  This is the amount you have chosen to live in excellent health. You are giving up the rest of the 60 years.

______   Numbers of years  (1)

Q16 Imagine that you are in Health State B and you have 60 years left to live. We are interested to know whether you would be prepared to live for less than 60 years if your health could be excellent as in Health State A.  Please slide the marker to the shortest time you would accept Health State A instead of 60 years in Health State B.  This is the amount you have chosen to live in excellent health. You are giving up the rest of the 60 years.

______   Numbers of years  (1)

Q17a We'd like to know how difficult you found the last section.Overall, I found the last section:

- Very easy (1)
- Neither too easy but not too difficult (2)
- Very difficult (3)

Q17b Did you need help with the last section?

- Yes (1)
- No (2)

Q17c Write any comments you may have about the last section here:

Q17d If you needed help with the last section, who helped you?

- Parent (1)
- Brother or sister (2)
- Friend (3)
- Other (4)

Q19 InstructionsWe'd like to know how you feel about Health State A compared to other health states. In this section, please:1. Imagine that ... You could live in the described health state for 60 more years.  OR  You could live in Health State A, but there is a risk of living in Health State D. 2. Check the box indicating whether you would prefer to live in the described health state, or take the chance of living in Health State A with the risk of living in Health State D.

Q20 Imagine that you are living in Health State C. There is new medicine that will let you live in Health State A. But the medicine isn&#39;t perfect and there is a chance that you could end up living in Health State D.  We are interested to know if you would chose to live in Health State C, or take the medicine with a 0% chance of living in Health State A and a 100% chance of living in Health State D.

- Live in Health State C (1)
- Take the medicine with a 0% chance of living in Health State A and 100% chance of living in Health State D (2)

Q23 Imagine that you are living in Health State C. There is new medicine that will let you live in Health State A. But the medicine isn&#39;t perfect and there is a chance that you could end up living in Health State D.  We are interested to know if you would chose to live in Health State C, or take the medicine with a 30% chance of living in Health State A and a 70% chance of living in Health State D.

- Live in Health State C (1)
- Take the medicine with a 30% chance of living in Health State A and 70% chance of living in Health State D (2)

Q25 Imagine that you are living in Health State C. There is new medicine that will let you live in Health State A. But the medicine isn&#39;t perfect and there is a chance that you could end up living in Health State D.  We are interested to know if you would chose to live in Health State C, or take the medicine with a 50% chance of living in Health State A and a 50% chance of living in Health State D.

- Live in Health State C (1)
- Take the medicine with a 50% chance of living in Health State A and 50% chance of living in Health State D (2)

Q29   Imagine that you are living in Health State C. There is new medicine that will let you live in Health State A. But the medicine isn&#39;t perfect and there is a chance that you could end up living in Health State D.  We are interested to know if you would chose to live in Health State C, or take the medicine with a 90% chance of living in Health State A and a 10% chance of living in Health State D.

- Live in Health State C (1)
- Take the medicine with a 90% chance of living in Health State A and 10% chance of living in Health State D (2)

Q30   Imagine that you are living in Health State C. There is new medicine that will let you live in Health State A. But the medicine isn&#39;t perfect and there is a chance that you could end up living in Health State D.  We are interested to know if you would chose to live in Health State C, or take the medicine with a 100% chance of living in Health State A and a 0% chance of living in Health State D.

- Live in Health State C (1)
- Take the medicine with a 100% chance of living in Health State A and 0% chance of living in Health State D (2)

Q31 Now let's try it with Health State B.

Q32 Imagine that you are living in Health State B. There is new medicine that will let you live in Health State A. But the medicine isn&#39;t perfect and there is a chance that you could end up living in Health State D.  We are interested to know if you would chose to live in Health State B, or take the medicine with a 0% chance of living in Health State A and a 100% chance of living in Health State D.

- Live in Health State B (1)
- Take the medicine with a 0% chance of living in Health State A and 100% chance of living in Health State D (2)

Q35 Imagine that you are living in Health State B. There is new medicine that will let you live in Health State A. But the medicine isn&#39;t perfect and there is a chance that you could end up living in Health State D.  We are interested to know if you would chose to live in Health State B, or take the medicine with a 30% chance of living in Health State A and a 70% chance of living in Health State D.

- Live in Health State B (1)
- Take the medicine with a 30% chance of living in Health State A and 70% chance of living in Health State D (2)

Q37   Imagine that you are living in Health State B. There is new medicine that will let you live in Health State A. But the medicine isn&#39;t perfect and there is a chance that you could end up living in Health State D.  We are interested to know if you would chose to live in Health State B, or take the medicine with a 50% chance of living in Health State A and a 50% chance of living in Health State D.

- Live in Health State B (1)
- Take the medicine with a 50% chance of living in Health State A and 50% chance of living in Health State D (2)

Q41 Imagine that you are living in Health State B. There is new medicine that will let you live in Health State A. But the medicine isn&#39;t perfect and there is a chance that you could end up living in Health State D.  We are interested to know if you would chose to live in Health State B, or take the medicine with a 90% chance of living in Health State A and a 10% chance of living in Health State D.

- Live in Health State B (1)
- Take the medicine with a 90% chance of living in Health State A and 10% chance of living in Health State D (2)

Q42 Imagine that you are living in Health State B. There is new medicine that will let you live in Health State A. But the medicine isn&#39;t perfect and there is a chance that you could end up living in Health State D.  We are interested to know if you would chose to live in Health State B, or take the medicine with a 100% chance of living in Health State A and a 0% chance of living in Health State D.

- Live in Health State B (1)
- Take the medicine with a 100% chance of living in Health State A and 0% chance of living in Health State D (2)

Q43a We'd like to know how difficult you found the last section. Overall, I found the last section:

- Very easy (1)
- Neither too easy but not too difficult (2)
- Very difficult (3)

Q43b Did you need help with the last section?

- Yes (1)
- No (2)

Q43c Write any comments you may have about the last section here:

Q43d If you needed help with the last section, who helped you?

- Parent (1)
- Brother or sister (2)
- Friend (3)
- Other (4)

Q52 Now we have a few questions about you. How old are you today?

- 10 years old (1)
- 11 years old (2)
- 12 years old (3)
- 13 years old (4)
- 14 years old (5)
- 15 years old (6)
- 16 years old (7)
- 17 years old (8)
- 18 years old (9)
- 19 years old (10)
- 20 years old (11)
- 21 years old (12)

Q53 What grade are you currently in at school?

- 4th grade (1)
- 5th grade (2)
- 6th grade (3)
- 7th grade (4)
- 8th grade (5)
- 9th grade (6)
- 10th grade (7)
- 11th grade (8)
- 12th grade (9)
- Other (10)

Q53a Please specify what grade you are currently in?

Q82 Thank you for completing the survey. For testing purposes, we&#39;d like to know if you&#39;d be willing to re-take this survey in a week&#39;s time. If you choose to do this, your responses between the two surveys will be compared. Would you be willing to do this survey again?

- Yes (1)
- No (2)
